# Next generation long-culm rice with superior lodging resistance and high grain yield, Monster Rice 1

**DOI:** 10.1371/journal.pone.0221424

**Published:** 2019-08-22

**Authors:** Tomohiro Nomura, Naoya Arakawa, Toshio Yamamoto, Tadamasa Ueda, Shunsuke Adachi, Jun-ichi Yonemaru, Akira Abe, Hiroki Takagi, Tadashi Yokoyama, Taiichiro Ookawa

**Affiliations:** 1 Graduate School of Agriculture, Tokyo University of Agriculture and Technology, Fuchu, Tokyo, Japan; 2 Institute of Plant Science and Resources, Okayama University, Kurashiki, Okayama, Japan; 3 Institute of Crop Science, NARO, Tsukuba, Ibaraki, Japan; 4 Department of Genomics and Breeding, Iwate Biotechnology Research Center, Kitakami, Iwate, Japan; 5 Faculty of Bioresources and Environmental Sciences, Ishikawa Prefectural University, Nonoichi, Ishikawa, Japan; International Rice Research Institute, PHILIPPINES

## Abstract

During late 1960s Green Revolution, researchers utilized *semidwarf 1* (*sd1*) to improve the yield and lodging resistance in rice (*Oryza sativa* L.). However, *sd1* has a negative effect to culm strength and biomass production. To increase yield dramatically in 21^th^ century, development of next generation long-culm rice for non-lodging and high grain yield independent of *sd1* has been needed. The present study developed Monster Rice 1, a long-culm and heavy-panicle type of rice line and compared it with Takanari, a high-yielding semidwarf rice variety about yield and lodging resistance associated traits. Brown rice yield and bending moment at breaking of the basal elongated internode were higher in Monster Rice 1 than those in Takanari due to a large number of spikelets per panicle and thicker culm. Furthermore, to identify QTLs with superior alleles for these traits, QTL and haplotype analyses were performed using F_2_ population and recombinant inbred lines derived from a cross between Monster Rice 1 and Takanari. The results from this study suggest that long-culm and heavy-panicle type of rice with a superior lodging resistance by culm strength can perform its high yield potential by using these identified QTLs contributing yield and lodging resistance.

## Introduction

Through simulation model up to 2050, the world population may be expected to reach about 9 billion human population, and it is necessary to increase the crop yield to meet the growing food demand in same ratio [1]. Rice (*Oryza sativa* L.) is one of the most important crops—it accounts for over 21% of the caloric needs of the world’s population and 76% of the caloric intake of Southeast Asia [2]. During late 1960s, rice harvested yield was dramatically increased by semidwarf varieties by gene introgression of *semidwarf 1* (*sd1*), here the harvest index increased with non-lodging under a significant amount of fertilizer application. This was known as the “Green Revolution” [3]. However, it has been pointed out that the biomass production potential of semidwarf varieties was lower than that in long-culm varieties, because leaf area density in the canopy was high and CO_2_ diffusion efficiency was poor due to low plant height [4,5]. Furthermore, gibberellin-deficient semidwarf mutants such as *sd1* negatively affect not only biomass production but also grain weight as compared with wild types [6]. Therefore, further increases in yield require the use of long-culm and high biomass rice varieties with a strong culm that resists lodging. In addition, the intensities of typhoons hitting East and Southeast Asia have increased due to ocean surface warming and may be likely to increase further [7]. Therefore, lodging resistance with strong culm is one of the primary breeding objectives.

In order to breed this type of rice varieties, quantitative trait loci (QTLs) associated with a strong culm should be identified. *STRONG CULM 1* (*SCM1*) and *STRONG CULM2* (*SCM2*) have been identified, using chromosome segment substitution lines from a cross between the *indica* variety Habataki and the *japonica* variety Sasanishiki. The responsive gene of *SCM2* was identified as *ABERRANT PANICLE ORGANIZATION 1* (*APO1*) [8,9]. *STRONG CULM 3* (*SCM3*) and *STRONG CULM4* (*SCM4*) have also been identified using backcross inbred lines derived from a cross between the *tropical japonica* variety Chugoku 117 [10] and the *japonica* variety Koshihikari. The responsive gene of *SCM3* was identified as *TEOSINTE BRANCHED 1* (*OsTB1*)/*FINE CULM1* (*FC1*) [11–13]. In addition, the QTLs *prl5* [14], *lrt5* [15], *BSUC11* [16,17] and *SD1* [18] have been found to be related to strong culm.

The current study developed new rice lines derived from crosses among varieties with superior alleles associated with traits for high yield and lodging resistance. Initially, TULT-gh-5-5 was selected from a cross between long- and strong-culm and high biomass variety Leaf Star with superior *SCM3* and *SCM4* alleles originating from Chugoku 117 [19,13] and the high-yielding variety Takanari with superior *SCM1* and *SCM2* alleles [9]. TUAT-32HB was selected from a cross between high-yielding varieties Akenohoshi and Takanari. Furthermore, the very long-culm, super thick-culm and super grain-bearing line, Monster Rice 1 was derived from a cross between TULT-gh-5-5 and TUAT-32HB. However, details of biomass production, yield and lodging resistance of Monster Rice 1 have not been clarified to date.

The current study investigated the characteristics of Monster Rice 1 by comparing with the high-yielding, high lodging-resistant and semidwarf variety Takanari. Monster Rice 1 was found to have long and large panicles and thick culms. Then, the current study identified the QTLs for these traits, using progenies of Monster Rice 1 and Takanari crossing. Furthermore, the current study surveyed the combined effect of QTLs for culm thickness using recombinant inbred lines (RILs) to develop the breeding lines with further high yield and lodging resistance.

## Materials and methods

### Plant materials and cultivation

**Fig 1:**
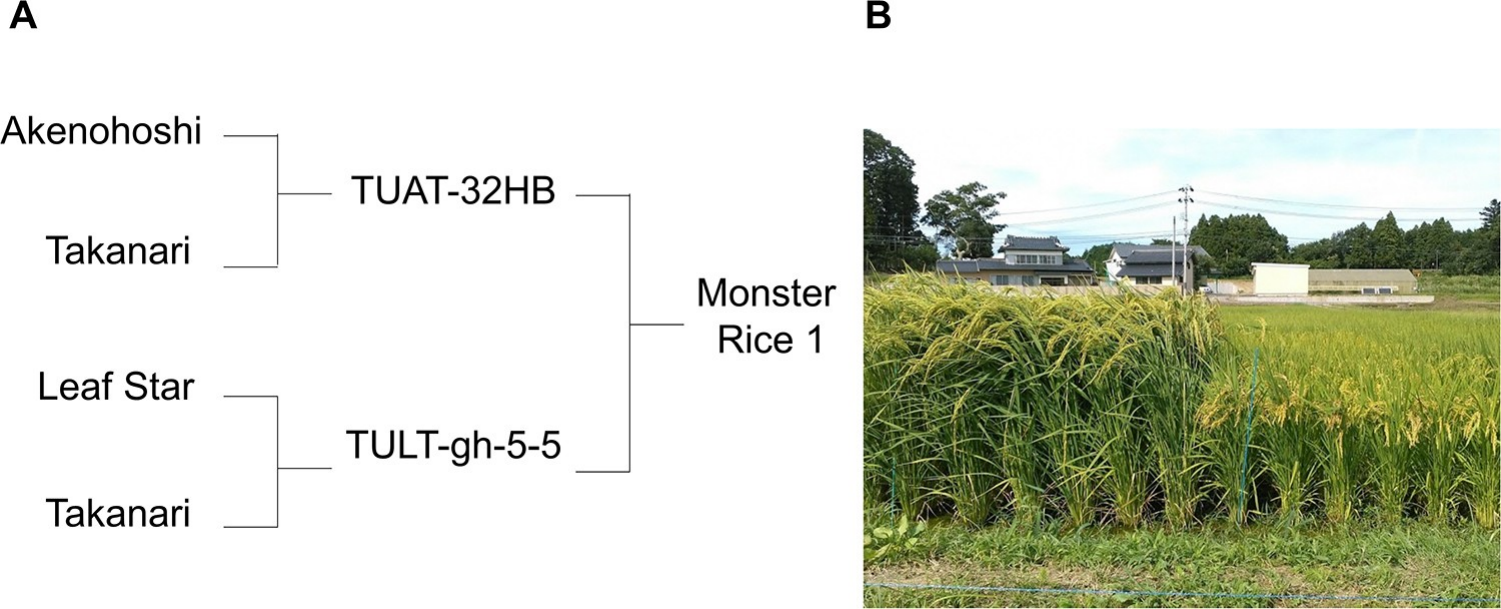
The pedigree chart and canopy of Monster Rice 1. (A) The pedigree chart of Monster Rice 1. (B) Left canopy is Monster Rice 1 and right canopy is Fukuhibiki, which is a high-yielding rice variety in Fukushima, Japan.

The rice (*Oryza sativa* L.) line and cultivar, Monster Rice 1 and Takanari were compared in 2016 and 2017. As mentioned above, Monster Rice 1 has been developed from crosses among varieties with superior alleles associated with traits for high yield and lodging resistance (Fig 1) and belongs to a group of moderate maturation. On the other hand, Takanari is a high-yielding, high lodging-resistant, and semidwarf *indica* variety released in 1990 and its suitable regions for cultivation are west of Kanto and Tokai in Japan (due to weak cold weather resistance) [20,21]. Takanari belongs to a group of moderate maturation and extra-heavy-panicle type [20,21]. Their F_2_ population (*N* = 94) and RILs (F_6_) (*N* = 94) were analyzed in 2015 and 2017, respectively. Field experiments were carried out on the Experimental Farm of Field Science Center, Tokyo University of Agriculture and Technology in 2015, 2016 and 2017. Because the cultivation methods were similar in these three years, the current study only described those used in 2017.

Seeds were sown in nursery boxes on 8 May 2017. Seedlings at the fourth leaf stage were transplanted to a paddy field on Tama River alluvial soil, using one seedling per hill on 25 May 2017. However, experiments on biomass and yield components were conducted using three seedlings per hill. The planting density was 22.2 hills m^-2^, with a spacing of 15 cm × 30 cm. N, P_2_O_5_ and K_2_O were applied at 50 kg ha^-1^, 60 kg ha^-1^ and 60 kg ha^-1^, respectively, as a basal fertilization. The field was submerged throughout the experiments by irrigation. Weed and pest controls were carried out as required.

Meteorological data sets including mean air temperature, precipitation, and sunlight duration during the growing period for three years were obtained from the Automated Meteorological Data Acquisition System (AMeDAS, http://www.jma.go.jp/jp/amedas/) in Fuchu Station (S1 Fig).

### Phenotyping

#### Evaluation of biomass traits

Stem and leaf, panicle, and plant weight (aboveground parts only) at harvest time were evaluated. For yield traits, three plants with average growth from each plot were bulked, oven dried at 80°C for 72 hours, and weighed.

#### Evaluation of panicle traits

Panicles of the main culms were sampled 15 days after heading. Number of primary branches, number of secondary branches, number of tertiary branches and number of spikelets were identified.

#### Evaluation of lodging resistance traits

Twenty-four main culms were sampled from each plot 15 days after heading, and 8 main culms with average basal elongated internode lengths were selected for measurements. Evaluations of lodging resistance used a Tensilon RTG-1210 universal testing machine (A&D, Tokyo, Japan) and were carried out by placing the basal elongated internode on fulcrums 4 cm in distance, and loading the center of the internode as described previously [22]. Physical parameters for breaking-type lodging resistance were calculated by the following formula.

M=σZ (1)

$$Z=\frac{\pi ({a_{1}}^{3}b_{1}-{a_{2}}^{3}b_{2})}{32a_{1}}$$ (2)

Physical parameters for bending-type lodging resistance were calculated by the following formula.

$$E=\frac{Pl^{3}}{48\delta I}$$ (3)

$$I=\frac{\pi ({a_{1}}^{3}b_{1}-{a_{2}}^{3}b_{2})}{64}$$ (4)

*M* is the bending moment at breaking, *σ* is the bending stress and *Z* is the section modulus. *a*_1_ is the outer diameter of the minor axis in an oval cross-section, *b*_1_ is the outer diameter of the major axis in an oval cross-section, *a*_2_ is the inner diameter of the minor axis in an oval cross-section, and *b*_2_ is the inner diameter of the major axis in an oval cross-section. *E* is the Young’s modulus, *P* is the load, *l* is the fulcrum distance, *δ* is the deflection, *I* is the secondary moment of inertia and *EI* is the flexural rigidity [22].

Cross sections of basal elongated internodes were stained with toluidine blue and photographed using a SZX 12, stereoscopic microscope (OLYMPUS, Tokyo, Japan).

### QTL analysis using F_2_ and RILs

For analysis of genotype, 151 single nucleotide polymorphism (SNP) markers for F_2_ population were calculated on a Golden Gate BeadArray technology platform (Illumina, San Diego, CA, USA) (S1 File). Eighty-seven Fluidigm (Fluidigm, San Francisco, CA, USA) assays for RILs were designed based on 151 polymorphisms identified by the Golden Gate BeadAssay technology platform used for F_2_ population. Although the original assays focused on a limited DNA region, additional nine assays were designed based on the polymorphism data, which increased the targeted region. Finally, 81 SNP makers were used for QTL analysis in RILs (S2 File) because 10 markers did not have polymorphisms and 5 markers could not be discriminated.

Linkage maps were constructed using F_2_ and RILs, respectively. Linkage order and genetic distance of SNP markers were calculated using MAPMAKER/Exp 3.0 [23], and QTLs were detected by QTL Cartographer ver. 2.5, using the composite interval mapping (CIM) method [24]. The critical threshold values of the logarithm of the odds (LOD) score were calculated by conducting 1,000 permutation tests with a significance level at *P* = 0.05.

### Next-generation DNA sequencing for haplotype analysis

Total DNA was extracted from leaves of Monster Rice 1 and its progenitors (Akenohoshi, Leaf Star and Takanari) by the CTAB method [25]. Sequence reads were obtained by using Illumina HiSeq 2000 system (Illumina, San Diego, CA, USA). Low-quality bases and the adaptors in the sequence reads were trimmed with Trimmomatic software [26]. Trimmed reads were mapped to the Nipponbare reference sequence, IRGSP-1.0 using BWA software [27], sorted and indexed using SAMtools software [28]. To improve the raw alignment around insertion and deletion mutations (indels), local re-alignments were performed using GATK software [29]. Polymerase chain reaction (PCR) duplicates were removed using Picard software (http://broadinstitute.github.io/picard). SNPs and indels were identified individually for each sample using both SAMtools and GATK software.

### Statistical analysis

The significance of the difference for each trait value between Monster Rice 1 and Takanari was determined by t-test. To investigate the superior allele combination, RILs were classified by the genotypes of SNP markers nearest to the three QTLs detected for section modulus, and the significances of the difference for section modulus between the Monster Rice 1, Takanari, RILs with three positive alleles and RILs with three negative alleles were determined by Tukey-Kramer test. Statistical comparison above was conducted in R software ver. 3.2.4.

## Results

### Identification of component traits for high yield and strong culm in Monster Rice 1

**Fig 2:**
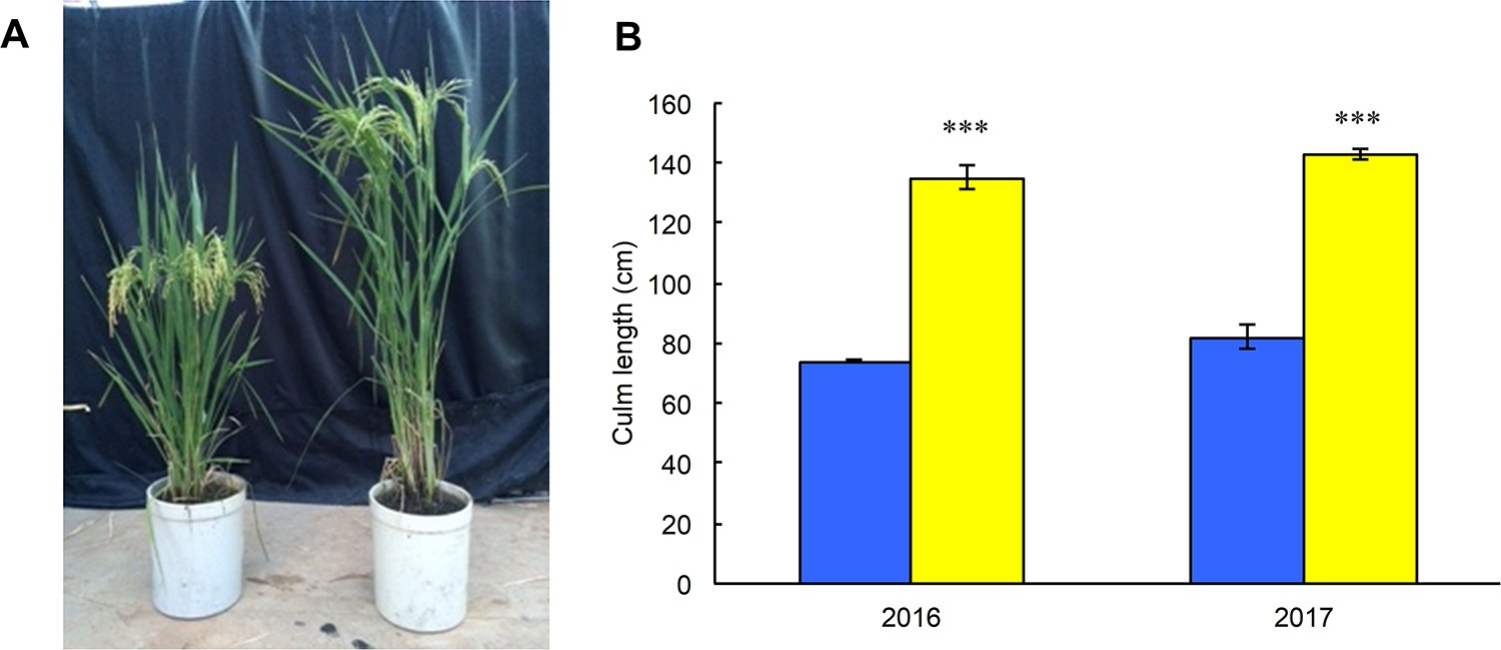
Plant appearance and culm length of Takanari and Monster Rice 1. (A) Plant appearance. Left and right plants indicate Takanari (a semidwarf variety) and Monster Rice 1 (an extra-long-culm line), respectively. (B) Culm length. Blue and yellow bars indicate mean ± SD (n = 3) of Takanari and Monster Rice 1, respectively. Asterisks indicate significant difference between both cultivars: *** indicates P < 0.001 (t-test).

To identify the component traits for high yield and strong culm, the current study compared yield and culm strength attributing traits between both parents. Mean culm length for Monster Rice 1 was 135 cm in 2016 and 143 cm in 2017, about 1.8 times higher than that of the short-culm variety, Takanari (Fig 2). This result indicated that Monster Rice 1 was an extra-long-culm line. The current study also compared dry matter production in Monster Rice 1 with that in Takanari (Table 1) and found that Monster Rice 1 showed significantly higher grain weight (*P* < 0.05) and plant weight (*P* < 0.05).

**Table 1:** Comparisons of biomass production between Monster Rice and Takanari in 2016.

	Stem and leaf weight	Grain weight	Plant weight
	(gDW m^-2^)	(gDW m^-2^)	(gDW m^-2^)
Monster Rice 1	995 ± 120	1036 ± 35	2031 ± 131
Takanari	763 ± 12	944 ± 19	1707 ± 19
	n.s.	*	*

Mean ± SD (n = 3). Asterisks indicate significant difference between cultivars: n.s. indicates no difference and * indicates *P* < 0.05 (t-test).

**Fig 3:**
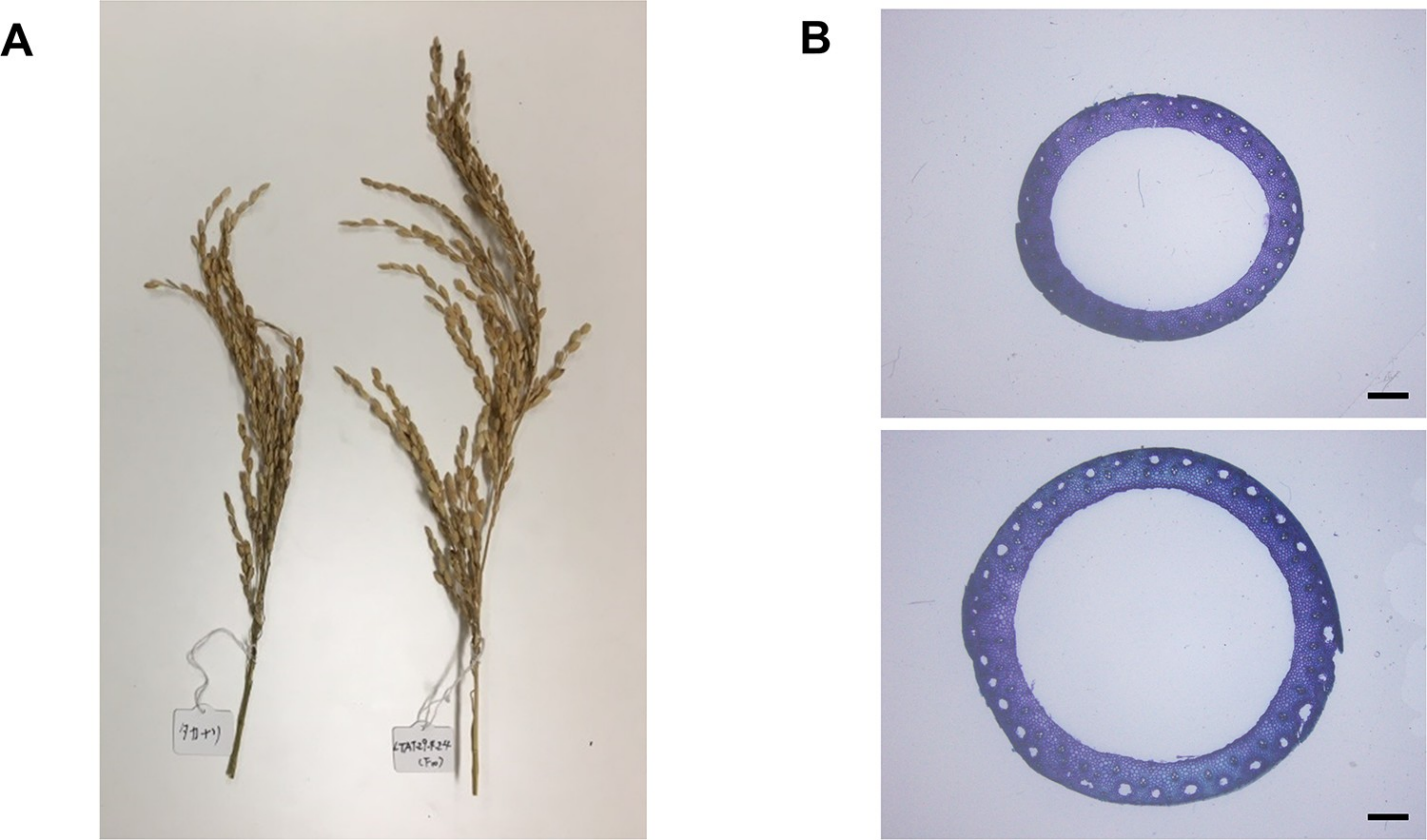
Panicle appearance and cross sections of basal elongated internodes. (A) Left and right panicles indicate Takanari and Monster Rice 1, respectively. (B) Upper and lower figures indicate cross sections of Takanari and Monster Rice 1, respectively. Black bar indicates 1 mm.

The current study further evaluated the yield from Monster Rice 1 by comparing the yield component between Monster Rice 1 and Takanari, a high-yielding variety (Table 2). Monster Rice 1 significantly decreased panicle number per square meter (*P* < 0.001). However, the yield in Monster Rice 1 significantly increased, due to the increase in the number of spikelets per panicle (*P* < 0.001). In fact, Monster Rice 1 showed a larger panicle appearance compared to Takanari (Fig 3A). On the other hand, there were no significant differences between the two varieties in the percentage of ripened grains and 1000-grain weight (Table 2).

**Table 2:** Comparisons of yield components between Monster Rice 1 and Takanari in 2016.

	Panicle number per m^2^	Number of spikelets per panicle	Percentage of ripened grains	1000-grain weight	Brown rice yield
	(Panicles m^-2^)	(Spikeles/Panicle)	(%)	(g)	(t ha^-1^)
**Monster Rice 1**	224.5 ± 4.3	252.3 ± 3.9	80.0 ± 1.1	20.0 ± 0.3	9.22 ± 0.26
**Takanari**	263.9 ± 4.3	194.5 ± 2.4	79.3 ± 1.0	19.8 ± 0.4	8.28 ± 0.12
**Monster Rice 1/Takanari**	0.85	1.30	1.01	1.01	1.11
	***	***	n.s.	n.s.	**

Mean ± SD (n = 3). Asterisks indicate significant difference between cultivars: n.s. indicates no difference, ** and *** indicate *P* < 0.01, 0.001, respectively (t-test).

To find component factors for large number of spikelets, the current study compared the number of branches and number of spikelets per branch between Monster Rice 1 and Takanari (Table 3). Primary branch number in Monster Rice 1 was significantly larger than that in Takanari (*P* < 0.05), but there was no significant difference in the number of spikelets of primary branches. The secondary branch number in Monster Rice 1 was about 1.6 times and significantly larger than that in Takanari (*P* < 0.001), and the number of the spikelets of the secondary branch was about 2.0 times and significantly larger (*P* < 0.001). In the tertiary branches, the average of Monster Rice 1 was 14.5, while the average of Takanari was 1.3. The number of spikelets of tertiary branches was significantly larger in Monster Rice 1 than that in Takanari (*P* < 0.001).

**Table 3:** Comparisons of branch and spikelet number per panicle in the main culm between Monster Rice 1 and Takanari in 2017.

	Branch number per panicle			Spikelet number per panicle			
	Primary branches	Secondary branches	Tertiary branches	Spikelets of primary branches	Spikelets of secondary branches	Spikelets of tertiary branches	Total spikelets
**Monster Rice 1**	19.6 ± 1.0	70.7 ± 1.8	14.5 ± 2.7	98.3 ± 3.9	279.0 ± 8.0	36.3 ± 5.3	413.5 ± 16.3
**Takanari**	16.8 ± 0.4	42.9 ± 3.3	1.3 ± 0.8	92.5 ± 2.3	139.8 ± 11.1	2.7 ± 1.7	235.0 ± 12.5
	*	***	**	n.s	***	***	***

Mean ± SD (n = 3). Asterisks indicate significant difference between cultivars: n.s. indicates no difference, *, ** and *** indicate *P* < 0.05, 0.01, 0.001, respectively (t-test).

Next, we classified the traits for lodging resistance into the breaking type and the bending type, and compared these traits between Monster Rice 1 with Takanari (Table 4). In the breaking-type lodging resistance, bending moment at breaking was significantly larger in Monster Rice 1 in both 2016 (*P* < 0.001) and 2017 (*P* < 0.001). For component traits of bending moment at breaking, section modulus was more than two times and significantly larger in Monster Rice 1 than that in Takanari in both 2016 (*P* < 0.01) and 2017 (*P* < 0.01). While bending stress in Takanari was significantly larger than that in Monster Rice 1 in 2016 (*P* < 0.05), but there was no significant difference in 2017. Therefore, Monster Rice 1 showed a large bending moment at breaking due to its high section modulus.

**Table 4:** Comparisons of the component traits of lodging resistance between Monster Rice 1 and Takanari in 2016 and 2017.

Year	Line	The component traits of breaking-type lodging resistance			The component traits of bending-type lodging resistance		
		Bending moment at breaking	Section modulus	Bending stress	Flexural rigidity	Secondary moment of inertia	Young's modulus
		(gf cm)	(mm^3^)	(gf mm^-2^)	(kgf cm^2^)	(mm^4^)	(kgf mm^-2^)
2016	Monster Rice 1	2456±167	31.4±3.2	793±72	17.9±1.5	120±15	15.7±2.4
	Takanari	1412±99	15.1±0.7	950±65	15.4±1.2	44.0±1.9	35.8±1.9
		***	**	*	n.s.	*	***
2017	Monster Rice 1	2731±79	44.4±0.3	615±18	18.4±0.8	186±5	9.97±0.74
	Takanari	1276±173	19.7±1.9	654±42	12.9±2.2	61.8±7.4	21.3±1.9
		***	**	n.s.	*	***	**

Mean ± SD (n = 3, except for flexural rigidity and Young’s modulus of Monster Rice 1 in 2017, n = 2). Asterisks indicate significant difference between cultivars: n.s. indicates no difference, *, ** and *** indicate *P* < 0.05, 0.01, 0.001, respectively (t-test).

In the bending-type lodging resistance, flexural rigidity was not significantly different in 2016, but Monster Rice 1 was significantly larger than Takanari in 2017 (*P* < 0.05). For the component traits of flexural rigidity, secondary moment of inertia in Monster Rice 1 was significantly larger than in Takanari in both 2016 (*P* < 0.05) and 2017 (*P* < 0.001). On the other hand, Young's modulus in Monster Rice 1 was significantly smaller than in Takanari in both 2016 (*P* < 0.001) and 2017 (*P* < 0.01).

The current study also observed the cross section of the internodes using a stereoscopic microscope. Monster Rice 1 has a large, thick cross section, compared with that of Takanari (Fig 3B). These results indicated that Monster Rice 1 had an extra thick culm.

### QTLs for yield and lodging resistance

The current study researched frequency distributions for the traits associated with yield and lodging resistance in F_2_ population and RILs (S2 and S3 Figs). Both populations were continuously distributed for all evaluated traits, and there were transgressive segregations for some traits. This result indicated that multiple QTLs contribute to the traits in F_2_ population and RILs. Then, to clarify the QTLs for the traits in Monster Rice 1, the current study created a linkage map (S4 and S5 Figs), and performed QTL analysis for these component traits, except for bending-type lodging resistance (Tables 5 and 6). In F_2_ populations (*N* = 94), four QTLs for branch or spikelet number and three QTLs for lodging resistance were detected on Chromosomes (Chrs.) 1, 2, 4 and 12. In RILs population (*N* = 94), 17 QTLs for branch or spikelet number, and 6 QTLs for lodging resistance were detected on Chr.1, 4, 7, 9, 10 and 12.

**Table 5:** Summary of QTLs detected in the F_2_ and recombinant inbred lines from a cross between Monster Rice 1 and Takanari about rachis branching and spikelet number per panicle.

	Trait	Chromosome	Position (cM)	Left marker	Right marker	Nearest marker	Position (Mb)	LOD	Additive effect	PVE (%)
**F**_**2**_	**Secondary branch number**	1	90	P0669	ah01002411	P0669	33.03	3.32	3.3	11.0
	**Secondary branch number**	2	61	P0221_3	ah02001353	P1287	14.54	3.74	-5.3	14.1
	**Secondary branch number**	4*	132	P0732	ad04012391	P0738	31.61	3.77	5.6	14.2
	**Spikelet number of secondary branch**	2	46	P0221_3	AE02002124	ah02000807	12.25	3.64	-53.4	39.8
**RILs**	**Primary brach number**	4	212	FA1017	FA1048	FA1040	25.90	8.10	0.9	28.0
	**Primary brach number**	12	0	-	FA1770	FA1764	25.53	2.86	0.5	8.4
	**Secondary branch number**	1	60	FA0731	FA0749	FA0731	32.70	3.28	3.8	21.5
	**Secondary branch number**	4	9	FA1001	FA1012	FA1001	12.70	2.45	-2.2	7.8
	**Secondary branch number**	4*	223	FA1017	FA1052	FA1048	30.69	7.19	4.3	27.9
	**Secondary branch number**	9	43	FA1425	FA1439	FA1439	16.80	3.45	4.4	31.7
	**Secondary branch number**	10	165	FA1457	FA1498	FA1498	13.30	2.57	4.7	34.6
	**Spikelet number of primary branch**	7	5	FA1218	FA1225	FA1221	1.45	3.22	-4.2	11.7
	**Spikelet number of primary branch**	12	9	-	-	FA1775	27.47	3.95	4.7	13.5
	**Spikelet number of secondary branch**	4	232	FA1046	FA1060	FA1052	31.66	9.17	19.5	32.0
	**Spikelet number of secondary branch**	7	56	FA1225	-	FA1284	22.82	5.11	20.7	37.0
	**Spikelet number of secondary branch**	9	72	FA1425	FA1445	FA1442	17.89	4.95	20.1	17.0
	**Spikelet number of tertiary branch**	4	234	FA1052	FA1055	FA1055	32.60	2.32	2.4	10.4
	**Total spiketet number**	4	232	FA1046	FA1060	FA1052	31.66	9.77	23.1	33.3
	**Total spiketet number**	7	55	FA1225	-	FA1284	22.82	5.48	24.3	38.5
	**Total spiketet number**	9	62	FA1425	FA1442	FA1442	17.89	4.22	24.3	30.1
	**Total spiketet number**	9	84	FA1448	-	FA1448	19.84	3.24	-17.9	9.6

Each physical distance represents nearest marker positions in the QTLs based on the rice genome (Build 4 for F_2_ population and IRGSP-1.0 for RILs, http://rapdb.dna.affrc.go.jp/) of the cultivar 'Nipponbare' (*O*. *sativa* ssp. *japonica*). The additive effect indicates in the insertion of the Monster Rice 1 allele. The QTLs detected by 1000 permutation tests with a significance level of 5% is shown. Asterisks indicate the QTLs detected in both F_2_ and RILs. Spikelet number of tertiary branch was counted as spikelet number of secondary branch only in F_2_.

**Table 6:** Summary of QTLs detected in the F_2_ and recombinant inbred lines from a cross between Monster Rice 1 and Takanari about breaking-type lodging resistance.

	Trait	Chromosome	Position (cM)	Left marker	Right marker	Nearest marker	Position (Mb)	LOD	Additive effect	PVE (%)
**F**_**2**_	**Section modulus**	1*	116	P0686	ah01003209	ah01003105	40.25	4.24	4.51	14.1
	**Section modulus**	2	151	AE02004954	ad02017623	P0269	34.89	3.66	3.89	12.5
	**Bending stress**	12*	33	ad12009568	-	ad12009894	27.70	3.88	-91.8	22.4
**RILs**	**Section modulus**	1*	58	FA1902	FA0754	FA0731	32.70	7.36	4.66	32.7
	**Section modulus**	9	17	FA1425	FA1439	FA1425	10.34	2.81	2.44	8.9
	**Section modulus**	10	197	FA1457	FA1521	FA1505	16.24	3.99	-2.71	12.1
	**Bending stress**	1	64	FA0731	FA0751	FA0749	38.50	3.98	-62.5	22.3
	**Bending stress**	10	216	FA1516	FA1524	FA1521	20.84	4.19	58.4	15.1
	**Bending stress**	12*	0	-	FA1770	FA1764	25.53	2.76	-37.5	9.3

Each physical distance represents nearest marker positions in the QTLs based on the rice genome (Build 4 for F_2_ population and IRGSP-1.0 for RILs, http://rapdb.dna.affrc.go.jp/) of the cultivar 'Nipponbare' (*O*. *sativa* ssp. *japonica*). The additive effect indicates in the insertion of the Monster Rice 1 allele. The QTLs detected by 1000 permutation tests with a significance level of 5% is shown. Asterisks indicate the QTLs detected in both F_2_ and RILs.

The QTL for spikelet number was detected at almost the same position in primary branch number, secondary branch number, spikelet number of the secondary branch, spikelet number of tertiary branch and total spikelet number at the long arm region on Chr.4 (Table 5). This QTL had a positive additive-effect on all these five traits, when the Monster Rice 1 allele was inserted into Takanari. In addition, in RILs, the QTL that increased secondary branch number, spikelet number of secondary branch and total spikelet number was detected on Chr.9 and the QTL that increased primary branch number and spikelet number of the primary branch was detected on Chr.12 by the insertion of the Monster Rice 1 allele into Takanari.

Focusing on the culm thickness associated with lodging resistance, QTLs for section modulus were detected on Chrs.1 and 2 in F_2_ (Table 6). In RILs, QTLs for section modulus were detected on Chrs.1, 9 and 10. These QTLs on Chrs.1 and 9 showed positive additive-effects as these alleles of Monster Rice 1 were inserted into Takanari. This QTL on Chr.1 in RILs was at almost the same position of the QTL detected in F_2_ and the additive effect and the proportion of variance were the highest in detected QTLs for section modulus. On the other hand, the QTL on Chr.10 had positive additive-effect upon inserting the Takanari allele into Monster Rice 1, despite the fact that the culm was thicker in Monster Rice 1 than that in Takanari.

Next, the current study focused on the culm stiffness, which is also trait associated with lodging resistance. QTLs for bending stress were detected on Chrs.1, 10 and 12 in RILs, and the QTL on Chr.12 was also detected at almost the same position in the F_2_ population (Table 6). Only QTL on Chr.10 had a positive additive-effect that increased bending stress, but QTLs on Chrs.1 and 12 had negative additive-effects upon insertion of the Monster Rice 1 allele into Takanari.

### Haplotype of Monster Rice 1

**Fig 4:**
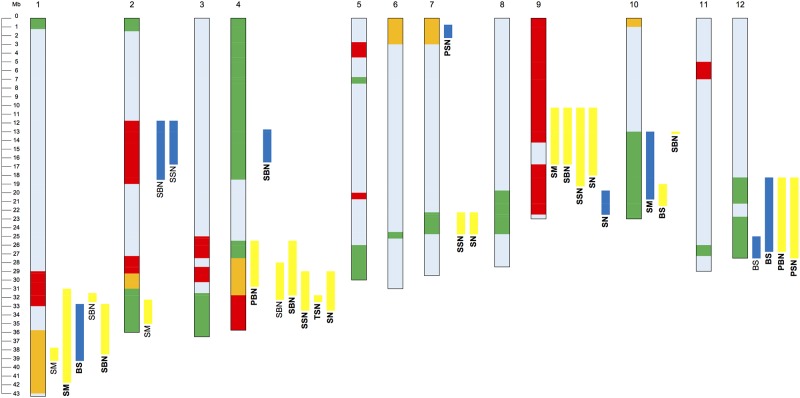
Haplotype of Monster Rice 1 and QTL positions. Green bar on chromosome, haplotype of Leaf Star; red bar on chromosome, haplotype of Akenohoshi; yellow bar on chromosome, same haplotype between Leaf Star and Akenohoshi; light blue bar on chromosome, Takanari haplotype. Yellow bar to the right of chromosome signifies a QTL for which Monster Rice 1 allele had a positive effect; blue bar to the right of chromosome signifies a QTL for which Takanari allele had a positive effect. Normal letters signify a QTL detected in F_2_; bold letters signify a QTL detected in RILs. SM, section modulus; BS, bending stress; PBN, primary branch number; SBN, secondary branch number; PSN, spikelet number of primary branch; SSN, spikelet number of secondary branch; TSN, spikelet number of tertiary branch; SN, total spikelet number. Physical map position is based on the rice genome (IRGSP-1.0, http://rapdb.dna.affrc.go.jp/) of the cultivar 'Nipponbare' (*O*. *sativa* ssp. *japonica*).

Haplotype analysis was performed to clarify the origins of alleles of these QTLs detected in Monster Rice 1 (Fig 4). The genetic origins of the QTLs responsible for section modulus and bending stress on Chr.10 and for primary branch number, spikelet number of primary branch and bending stress on Chr.12 were identified as Leaf Star. The regions of the QTLs responsible for secondary branch number and spikelet number of the secondary branch on Chr.2 and for secondary branch number, spikelet number of the secondary branch, total spikelet number and section modulus on Chr.9 were the haplotype as Akenohoshi. The region of the QTL that increased the section modulus by inserting the allele of Monster Rice 1 into Takanari on Chr.1 included same haplotype as Leaf Star and Akenohoshi. The genetic origin of the QTL that was located at the long arm region on Chr.4 and increased branch and spikelet numbers by inserting the allele of Monster Rice 1 into Takanari included same haplotype as Leaf Star and Akenohoshi.

### Superior allele combination of Monster Rice 1 and Takanari for section modulus in RILs

**Fig 5:**
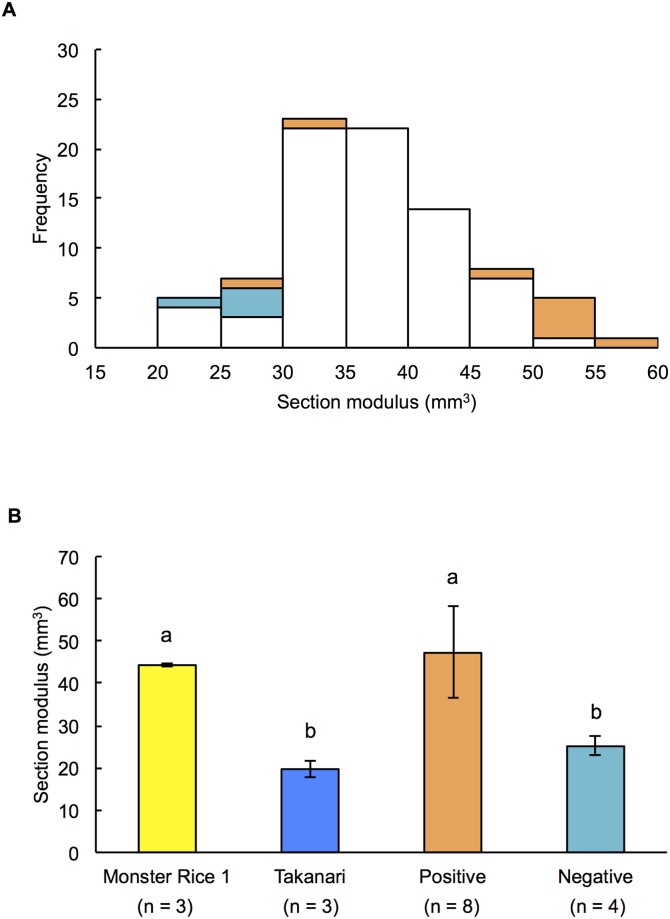
Combined effect of QTLs for section modulus in RILs. (A) Frequency distribution of section modulus. Orange bar, the lines combined with the three positive QTLs; light blue bar, the lines combined with three negative QTLs; white bar, the other lines. Blue and yellow arrows indicate mean of Takanari and Monster Rice 1, respectively. (B) Mean ± SD of section modulus of parental cultivars and lines with three positive or negative QTLs. Different alphabets indicate *P* < 0.05 (Tukey-Kramer test).

During RILs population investigation, the effect of superior allele combination of Monster Rice 1 and Takanari was found for the section modulus (Fig 5). Among 94 lines of RILs, lines were classified by the allele types using SNP markers nearest to the LOD peak of section modulus based on their genotypes (Fig 5A). Eight lines had the three alleles with a positive effect and three lines had three alleles with a negative effect. The mean value of the lines combined with the three positive alleles was higher than those of Takanari and the lines combined with the three negative alleles. On the other hand, the mean values were almost the same between the lines combined with three positive alleles and Monster Rice 1 (Fig 5B). This result indicated that these three positive alleles had a strong combined effect for section modulus.

## Discussion

### 1. Superior properties for a high yield and lodging resistance in new type variety, Monster Rice 1

The current study clarified the component traits associated with biomass production, yield and lodging resistance within developed RILs, Monster Rice 1, and detected the QTLs for these traits.

In a previous study, there is a high positive correlation between plant height and biomass [30]. However, in crop plants such as rice, although long-culm varieties had higher CO_2_ diffusion efficiency in the canopy due to lower leaf area density than short-culm varieties, there was not much difference in final biomass production due to the susceptibility to lodging [4,5]. In this study, the culm length of Monster Rice 1 was 1.8 times higher than that of the semidwarf variety, Takanari (Fig 2). However, Monster Rice 1 exhibited no lodging until the late ripening stage, despite its high plant height and heavy panicle, because Monster Rice 1 had high bending moment at breaking of the culm due to the section modulus, indicating a thick culm (Table 4, Fig 3B). In previous studies, lodging resistance was enhanced by thickening culms [13,31,32], but to our knowledge, there are no previous reports of varieties with such a large culm diameter (Fig 3B, S6 Fig). The current study results are consistent with a previous observation that long-culm varieties having a heavy panicle increased breaking resistance due to the increased culm thickness of the basal internode [33]. These results may suggest that during the long growth period, the canopy structure and high CO_2_ diffusion efficiency are maintained, which results in higher stem and leaf weight, grain weight and plant weight (Table 1). However, in the present study did not investigate canopy structure including leaf area density or gas diffusion inside the canopy and therefore further investigation is necessary in the future research.

For panicle, grain weight in Monster Rice 1 was 1.1 times higher than that in Takanari due to the bearing of a large panicle and 1.3 times larger number of spikelets per panicle (Tables 1 and 2, Fig 3A). It has been known that the number of spikelets per panicle had a trade-off relationship with panicle number [13]. Although a similar trade-off was also observed in this study, not only grain weight but also yield per hectare was significantly larger for Monster Rice 1 than Takanari (Table 2). Furthermore, the current study investigated the component traits of large number of spikelets in Monster Rice 1 in more detail. The current study found that the secondary branch number and tertiary branch number were particularly larger in Monster Rice 1 compared to Takanari, and also the spikelet numbers of the secondary and tertiary branches were larger. In addition to Takanari, previous studies have not reported the number of tertiary branches in Bekoaoba, Hokuriku 193 and Momiroman, which are the high-yielding varieties in Japan [34,35]. Therefore, the characteristic of the large number of tertiary branches of Monster Rice 1 may become one of the important traits that can be used for further breeding of high-yielding varieties.

### 2. QTLs for panicle branching in Monster Rice 1

The current study performed QTL analysis to investigate the genetic factors responsible for the component traits of rachis branching and spikelet number per panicle. In the QTL analysis by branch number, multiple QTLs that increased the number of secondary branches by inserting the allele of Monster Rice 1 into Takanari were detected, and one of these also increased the primary branch number (Table 5). This QTL was detected at the long arm region on Chr.4, and this haplotype was not distinguished between Leaf Star and Akenohoshi (Fig 4). The parents of Leaf Star are the *japonica* and *tropical japonica* varieties [19,10] and the progenitors of Akenohoshi include the *japonica* and *indica* varieties [36]. Previous studies have shown that the spikelet number increased as *NAL1* of the *japonica* type or *tropical japonica* type was introduced into the *indica* variety [37,38]. These results suggest that *NAL1* is a candidate gene for this detected QTL because of its functional effect, chromosomal position and haplotype.

In this study, QTLs for primary branch number and spikelet number of primary branches were detected on Chr.12 (Table 5). In addition, as a result of haplotype analysis, the origin of this QTL was identified as Leaf Star, derived from a cross between the *tropical japonica* variety, Chugoku 117 and the *japonica* variety, Koshihikari as mentioned above [19,10] (Fig 4). On the other hand, *qTSN12*.*1* and *qTSN12*.*2*, which are QTLs for total spikelet number per panicle, exist in this QTL region [39]. Sasaki et al. (2017) conducted an experiment using the near-isogenic line (NIL) of *indica* variety IR64 in which the *qTSN12*.*1* and *qTSN12*.*2* regions were replaced with YP 3 and YP 4, having a *tropical japonica* haplotype, although parents are different. As a result, both NILs exceeded the number of spikelets per panicle compared with IR64 [39]. These results support that the *indica* varieties in which this QTL region has been replaced with a *tropical japonica* variety, increase primary branch number and spikelet number on the primary branch. However, while *qTSN12*.*1* and *qTSN12*.*2* increase not only the number of primary branches but also the number of secondary branches [39], QTL on Chr.12 detected in the present study is a QTL only for the number of primary branches. Therefore, further examination is necessary in the future research.

The candidate genes for QTLs for panicle branching and spikelet number detected in this study include *APO2* [40] and *NAL1* on Chr.4. On the other hand, *LAX1* [41] presented near the QTL peak involved in the secondary branch detected on Chr.1, but haplotype analysis found that *LAX1* was a Takanari-type allele. Therefore, it is unlikely that *LAX1* is the responsible gene. In addition, *DEP1* [42] presents near the QTL peaks for secondary branch number, spikelet number of the secondary branch, total spikelet number on Chr.9. However, for the same reason as *LAX1*, the possibility that *DEP1* is the responsible gene is low. In the future, it will be necessary to clarify whether these candidate genes are the above-mentioned genes or novel genes. In addition, if these are novel genes, it will be important to clarify the physiological functions of the responsible genes.

### 3. QTLs for culm thickness and stiffness in Monster Rice 1

In this study, QTLs for culm thickness were detected on Chrs.1 and 2 in the F_2_ population and on Chrs.1, 9 and 10 in RILs (Table 6). The QTL on Chr.1 detected in both the F_2_ population and RILs contained the *SD1* region, and as a result of the haplotype analysis, this region of Monster Rice 1 indistinguishable from Leaf Star and Akenohoshi (Fig 4). As mentioned above, Leaf Star has alleles of the *japonica* and *tropical japonica* types [19,10], while Akenohoshi has alleles of the *japonica* and *indica* types [36]. This suggests that Monster Rice 1 has a *japonica* type functional *SD1*. On the other hand, Ookawa et al. (2016) demonstrated that the culm became thicker in the Takanari genetic background NIL, where *sd1* was replaced by *SD1* of the *japonica* variety, Koshihikari [18]. These results indicate that the culm of Monster Rice 1 is thicker than that in Takanari due to the effect of the *japonica* type functional *SD1*.

In previous researches, *SCM2/APO1* and *SCM3*/*FC1* were reported as the QTL that have the pleiotropic effects for culm strength and spikelet number [9,13]. In the present study, the QTL that increased section modulus by the insertion of the Monster Rice 1 allele into Takanari on Chr.9 also increased secondary branch number, spikelet number of secondary branch and total spikelet number (Tables 5 and 6). This result suggests that this QTL on Chr.9 also have the pleiotropic effects for culm strength and spikelet number.

The Monster Rice 1 QTL, which increased bending stress detected on Chr.10 in RILs, was a Leaf Star-type allele (Table 6, Fig 4). Leaf Star is known to have high mechanical strength because of high density biochemical cell wall components such as cellulose, hemicellulose, or lignin and thick cortical fiber tissue. However, the Leaf Star gene for high bending stress has been identified only as *OsCAD2* for lignin biosynthesis at the short arm region on Chr.2 [19,10]. Therefore, the QTL on Chr.10 detected in the present study, might be a novel QTL explaining the high bending stress of Leaf Star. However, further research will be needed to clarify how this QTL affects the culm anatomical traits.

### 4. Combined effects of QTLs for culm thickness

Finally, the current study surveyed the combined effects of QTLs for section modulus (Fig 5). In previous studies, target traits indicated characteristics similar or superior to those of their parents by pyramiding QTLs. For example, Matsubara et al. (2016) reported that lines that carried alleles with positive additive-effects on QTLs for plant weight were significantly higher than their parents [43]. It has also been reported that by pyramiding superior alleles for grain quality and grain yield of the parental varieties, both traits were expressed at the same level as in the parents simultaneously [44]. In the present study, although there was dispersion among the lines combined with three positive QTLs for section modulus, the average value of the section modulus of these lines was nearly identical to that of Monster Rice 1. These results suggest that by pyramiding the positive allele of Takanari in addition to the superior allele of Monster Rice 1, it is possible to breed lodging resistant rice varieties with a super thick-culm.

However, Monster Rice 1 still retains the problem of lodging after the late ripening stage due to its heavy panicle and biomass. In the future, similarly to Yano et al. (2015), who pyramided major QTLs, *SCM2*/*APO1* plus *SCM3*/*FC1* from two other varieties to strengthen the culm [13], it will be important to increase lodging resistance by further pyramiding QTLs for culm thickness and/or stiffness.

## Conclusions

Monster Rice 1, which is long-culm variety bred in this study, had high lodging resistance and yield, because the culm is much thicker and the grain number is larger than the high-yielding variety, Takanari. Furthermore, QTLs for those component traits were identified by QTL analysis using progenies of Monster Rice 1 and Takanari and the lines with QTLs that exhibit positive additive-effects showed the same culm thickness as Monster Rice 1. In addition to these QTLs, further pyramiding from other varieties suggests that the possibility of breeding new varieties that exhibit high yields and long culm but no lodging until harvest, may lead to the next green revolution.

## Supporting information

S1 Fig Meteorological conditions during the growing period for three years. (A) Mean air temperature and precipitation. Light blue marker, mean air temperature in 2015; orange marker, mean air temperature in 2016; gray marker, mean air temperature in 2017. Yellow bar, precipitation in 2015; blue bar, precipitation in 2016; green bar, precipitation in 2017. (B) Sunlight duration. Light blue marker, sunlight duration in 2015; orange marker, sunlight duration in 2016; gray marker, sunlight duration in 2017.
(PDF)

S2 Fig Frequency distribution for the traits associated with yield and lodging resistance in F_2_ population. (PDF)

S3 Fig Frequency distribution for the traits associated with yield and lodging resistance in RILs. Blue and yellow arrows indicate mean of Takanari and Monster Rice 1, respectively.
(PDF)

S4 Fig The linkage map of the F_2_ population. (PDF)

S5 Fig The linkage map of the RILs. (PDF)

S6 Fig Outer diameter of the major axis. Blue and yellow bars indicate mean ± SD (n = 3) of Takanari and Monster Rice 1, respectively. Asterisks indicate significant difference between both cultivars: *** indicates P < 0.001 (t-test).
(PDF)

S1 File Genotypic data of the F_2_ population. (XLSX)

S2 File Genotypic data of the RILs. (XLSX)

## References

[1] Tilman D, Balzer C, Hill J, Befort BL. (2011). Global food demand and the sustainable intensification of agriculture. Proc Natl Acad Sci U S A. 108: 20260–20264. 10.1073/pnas.1116437108 22106295 PMC3250154

[2] Fitzgerald MA, McCouch SR, Hall RD. (2009). Not just a grain of rice: the quest for quality. Trends Plant Sci. 14: 133–139. 10.1016/j.tplants.2008.12.004 19230745

[3] Khush GS. (1999). Green revolution: preparing for the 21st century. Genome. 42: 646–655. 10.1139/g99-044 10464789

[4] Takeda T, Oka M, Agata W. (1983). Characteristics of dry matter and grain production of rice cultivars in the Warmer Part of Japan: I. Comparison of dry matter production between old and new types of rice cultivars. Jpn J Crop Sci. 52: 299–306.

[5] Kuroda E, Ookawa T, Ishihara K. (1989). Analysis on difference of dry matter production between rice cultivars with different plant height in relation to gas diffusion inside stands. Jpn J Crop Sci. 58: 374–382.

[6] Okuno A, Hirano K, Asano K, Takase W, Masuda R, Morinaka Y, et al (2014). New approach to increasing rice lodging resistance and biomass yield through the use of high gibberellin producing varieties. PLoS ONE. 9: e86870 10.1371/journal.pone.0086870 24586255 PMC3929325

[7] Mei W, Xie SP. (2016). Intensification of landfalling typhoons over the northwest Pacific since the late 1970s. Nat Geosci. 9: 753–757. 10.1038/ngeo2792

[8] Ikeda-Kawakatsu K, Yasuno N, Oikawa T, Iida S, Nagato Y, Maekawa M, et al (2009). Expression level of *ABERRANT PANICLE ORGANIZATION 1* determines rice inflorescence form through control of cell proliferation in the meristem. Plant Physiol. 150: 736–747. 10.1104/pp.109.136739 19386809 PMC2689948

[9] Ookawa T, Hobo T, Yano M, Murata K, Ando T, Miura H, et al (2010). New approach for rice improvement using a pleiotropic QTL gene for lodging resistance and yield. Nat Commun. 1: 132 10.1038/ncomms1132 21119645 PMC3065348

[10] Ookawa T, Inoue K, Matsuoka M, Ebitani T, Takarada T, Yamamoto T, et al (2014). Increased lodging resistance in long-culm, low-lignin *gh2* rice for improved feed and bioenergy production. Sci Rep. 4: 6567 10.1038/srep06567 25298209 PMC4190510

[11] Takeda T, Suwa Y, Suzuki M, Kitano H, Ueguchi-Tanaka M, Ashikari M, et al (2003). The *OsTB1* gene negatively regulates lateral branching in rice. Plant J. 33: 513–520. 10.1046/j.1365-313X.2003.01648.x 12581309

[12] Minakuchi K, Kameoka H, Yasuno N, Umehara M, Luo L, Kobayashi K, et al (2010). *FINE CULM1* (*FC1*) works downstream of strigolactones to inhibit the outgrowth of axillary buds in rice. Plant Cell Physiol. 51: 1127–1135. 10.1093/pcp/pcq083 20547591 PMC2900823

[13] Yano K, Ookawa T, Aya K, Ochiai Y, Hirasawa T, Ebitani T, et al (2015). Isolation of a novel lodging resistance QTL gene involved in strigolactone signaling and its pyramiding with a QTL gene involved in another mechanism. Mol Plant. 8: 303–314. 10.1016/j.molp.2014.10.009 25616386

[14] Kashiwagi T, Ishimaru K. (2004). Identification and functional analysis of a locus for Improvement of lodging resistance in rice. Plant Physiol. 134: 676–683. 10.1104/pp.103.029355 14739343 PMC344543

[15] Ishimaru K, Togawa E, Ookawa T, Kashiwagi T, Madoka Y, Hirotsu N. (2008). New target for rice lodging resistance and its effect in a typhoon. Planta. 227: 601–609. 10.1007/s00425-007-0642-8 17960419

[16] Kashiwagi T. (2014). Identification of quantitative trait loci for resistance to bending-type lodging in rice (*Oryza sativa* L.). Euphytica. 198: 353–367. 10.1007/s10681-014-1111-7

[17] Kashiwagi T, Munakata J, Ishimaru K. (2016). Functional analysis of the lodging resistance QTL *BSUC11* on morphological and chemical characteristics in upper culms of rice. Euphytica. 210: 233–243. 10.1007/s10681-016-1707-1

[18] Ookawa T, Aoba R, Yamamoto T, Ueda T, Takai T, Fukuoka S, et al (2016). Precise estimation of genomic regions controlling lodging resistance using a set of reciprocal chromosome segment substitution lines in rice. Sci Rep. 6: 30572 10.1038/srep30572 27465821 PMC4964586

[19] Ookawa T, Yasuda K, Kato H, Sakai M, Seto M, Sunaga K, et al (2010). Biomass production and lodging resistance in ‘Leaf Star’, a new long-culm rice forage cultivar. Plant Prod Sci. 13: 58–66. 10.1626/pps.13.58

[20] Ando I. (1990). Breeding a new rice cultivar “Takanari”. Rep Kanto Br Crop Sci Soc Jpn. 5: 63–64.

[21] Imbe T, Akama Y, Nakane A, Hata T, Ise K, Ando I, et al (2004) Development of a multipurpose high-yielding rice variety “Takanari”. Bull Natl Inst Crop Sci. 5: 35–51.

[22] Ookawa T, Ishihara K. (1992). Varietal difference of physical characteristics of the culm related to lodging resistance in paddy rice. Jpn J Crop Sci. 61: 419–425.

[23] Lander ES, Green P, Abrahamson J, Barlow A, Daly MJ, Lincoln SE, et al (1987). MAPMAKER: An interactive computer package for constructing primary genetic linkage maps of experimental and natural populations. Genomics. 1: 174–181. 10.1016/0888-7543(87)90010-3 3692487

[24] Wang S, Basten CJ, Zeng ZB. (2012). Windows QTL Cartographer V2.5_011. Department of Statistics, North Carolina State University, Raleigh, NC. http://statgen.ncsu.edu/qtlcart/WQTLCart.htm

[25] Murray MG, Thompson WF. (1980). Rapid isolation of high molecular weight plant DNA. Nucleic Acids Res. 8: 4321–4325. 10.1093/nar/8.19.4321 7433111 PMC324241

[26] Bolger AM, Lohse M, Usadel B. (2014). Trimmomatic: a flexible trimmer for Illumina sequence data. Bioinformatics. 30: 2114–2120. 10.1093/bioinformatics/btu170 24695404 PMC4103590

[27] Li H, Durbin R. (2009). Fast and accurate short read alignment with Burrows-Wheeler transform. Bioinformatics. 25: 1754–1760. 10.1093/bioinformatics/btp324 19451168 PMC2705234

[28] Li H, Handsaker B, Wysoker A, Fennell T, Ruan J, Homer N, et al (2009). The Sequence Alignment/Map format and SAMtools. Bioinformatics. 25: 2078–2079. 10.1093/bioinformatics/btp352 19505943 PMC2723002

[29] DePristo MA, Banks E, Poplin R, Garimella KV, Maguire JR, Hartl C, et al (2011). A framework for variation discovery and genotyping using next-generation DNA sequencing data. Nat Genet. 43: 491–498. 10.1038/ng.806 21478889 PMC3083463

[30] Niklas KJ, Enquist BJ. (2001). Invariant scaling relationships for interspecific plant biomass production rates and body size. Proc Natl Acad Sci U S A. 98: 2922–2927. 10.1073/pnas.041590298 11226342 PMC30241

[31] Kashiwagi T, Togawa E, Hirotsu N, Ishimaru K. (2008). Improvement of lodging resistance with QTLs for stem diameter in rice (*Oryza sativa* L.). Theor Appl Genet. 117: 749–757. 10.1007/s00122-008-0816-1 18575836

[32] Hirano K, Okuno A, Hobo T, Ordonio R, Shinozaki Y, Asano K, et al (2014). Utilization of stiff culm trait of rice *smos1* mutant for increased lodging resistance. PLoS ONE. 9: e.96009 10.1371/journal.pone.0096009 24987959 PMC4079509

[33] Ma J, Ma W, Tian Y, Yang J, Zhou K, Zhu Q. (2004). The culm lodging resistance of heavy panicle type of rice. Acta Agron Sin. 30: 143–148.

[34] Yoshinaga S, Takai T, Arai-Sanoh Y, Ishimaru T, Kondo M. (2013). Varietal differences in sink production and grain-filling ability in recently developed high-yielding rice (*Oryza sativa* L.) varieties in Japan. Field Crops Res. 150: 74–82. 10.1016/j.fcr.2013.06.004

[35] Yabe S, Nakagawa H, Adachi S, Mukouyama T, Arai-Sanoh Y, Okamura M, et al (2018). Model analysis of genotypic difference in the variation of the duration from heading to flower opening based on the flower position on a panicle in high-yielding rice cultivars. Field Crops Res. 223: 155–163. 10.1016/j.fcr.2018.04.013

[36] Kaneda C. (1986). Rice breeding for extremely higher yielding ability by japonica-indica Hybridization. Jpn Agric Res Q. 19: 235–240

[37] Fujita D, Trijatmiko KR, Tagle AG, Sapasap MV, Koide Y, Sasaki K, et al (2013). *NAL1* allele from a rice landrace greatly increases yield in modern *indica* cultivars. Proc Natl Acad Sci U S A. 107: 20431–20436. 10.1073/pnas.1310790110 24297875 PMC3870739

[38] Zhang GH, Li SY, Wang L, Ye WJ, Zeng DL, Rao YC, et al (2014). *LSCHL4* from *japonica* cultivar, which is allelic to *NAL1*, increases yield of *indica* super rice 93–11. Mol Plant. 7: 1350–1364. 10.1093/mp/ssu055 24795339 PMC4115278

[39] Sasaki K, Fujita D, Koide Y, Lumanglas PD, Gannaban RB, Tagle AG, et al (2017). Fine mapping of a quantitative trait locus for spikelet number per panicle in a new plant type rice and evaluation of a near-isogenic line for grain productivity. J Exp Bot. 68: 2693–2702. 10.1093/jxb/erx128 28582550 PMC5853308

[40] Ikeda-kawakatsu K, Maekawa M, Izawa T, Itoh J, Nagato Y. (2012). *ABERRANT PANICLE ORGANIZATION 2*/*RFL*, the rice ortholog of Arabidopsis *LEAFY*, suppresses the transition from inflorescence meristem to floral meristem through interaction with *APO1*. Plant J. 69: 168–180. 10.1111/j.1365-313X.2011.04781.x 21910771

[41] Komatsu M, Maekawa M, Shimamoto K, Kyozuka J. (2001). The *LAX1* and *FRIZZY PANICLE 2* genes determine the inflorescence architecture of rice by controlling rachis-branch and spikelet development. Dev Biol. 231: 364–373. 10.1006/dbio.2000.9988 11237465

[42] Huang X, Qian Q, Liu Z, Sun H, He S, Luo D, et al (2009). Natural variation at the *DEP1* locus enhances grain yield in rice. Nat Genet. 41: 494–497. 10.1038/ng.352 19305410

[43] Matsubara K, Yamamoto E, Kobayashi N, Ishii T, Tanaka J, Tsunematsu H, et al (2016). Improvement of rice biomass yield through QTL-based selection. PLoS ONE. 11: e0151830 10.1371/journal.pone.0151830 26986071 PMC4795639

[44] Zeng D, Tian Z, Rao Y, Dong G, Yang Y, Huang L, et al (2017). Rational design of high-yield and superior-quality rice. Nat Plants. 3: 17031 10.1038/nplants.2017.31 28319055

