# Supplementary figures and images for: Next generation long-culm rice with superior lodging resistance and high grain yield, Monster Rice 1
Source: PLoS One. 2019 Aug 22;14(8):e0221424. doi: 10.1371/journal.pone.0221424 (PMC6705783; doi:10.1371/journal.pone.0221424)

**A**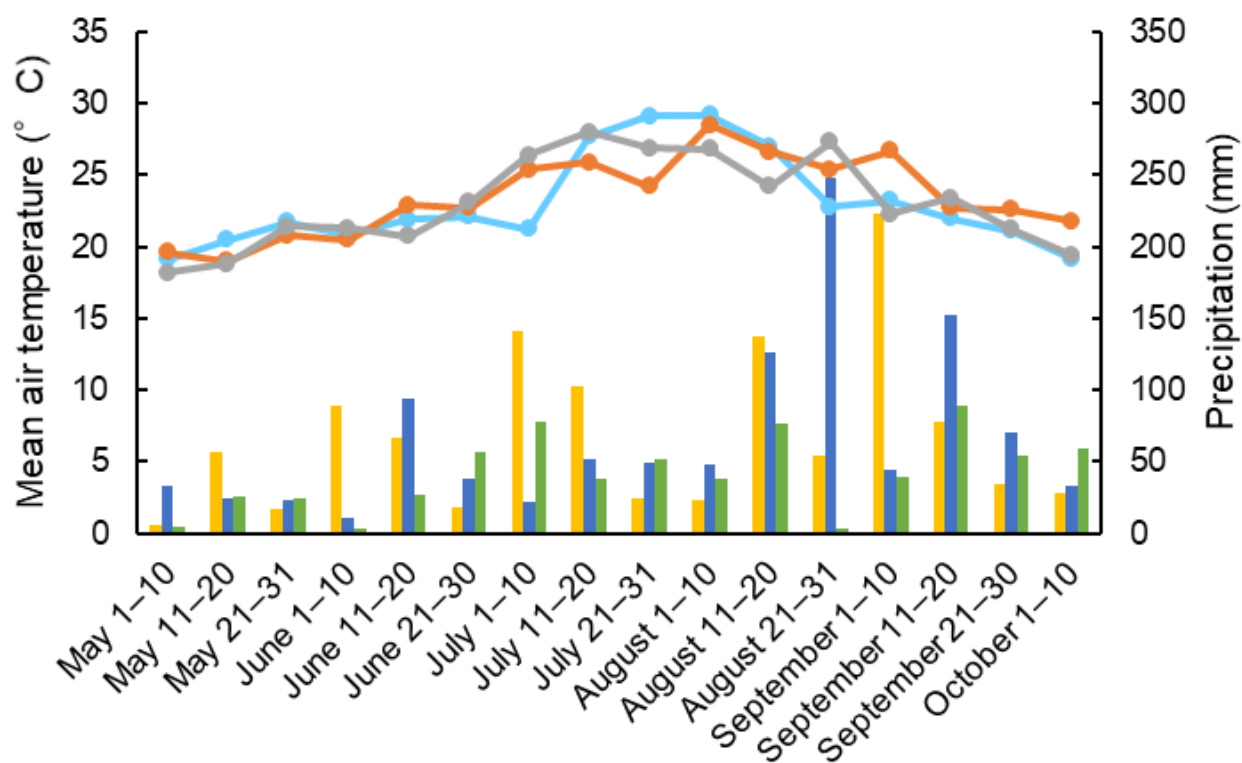**B**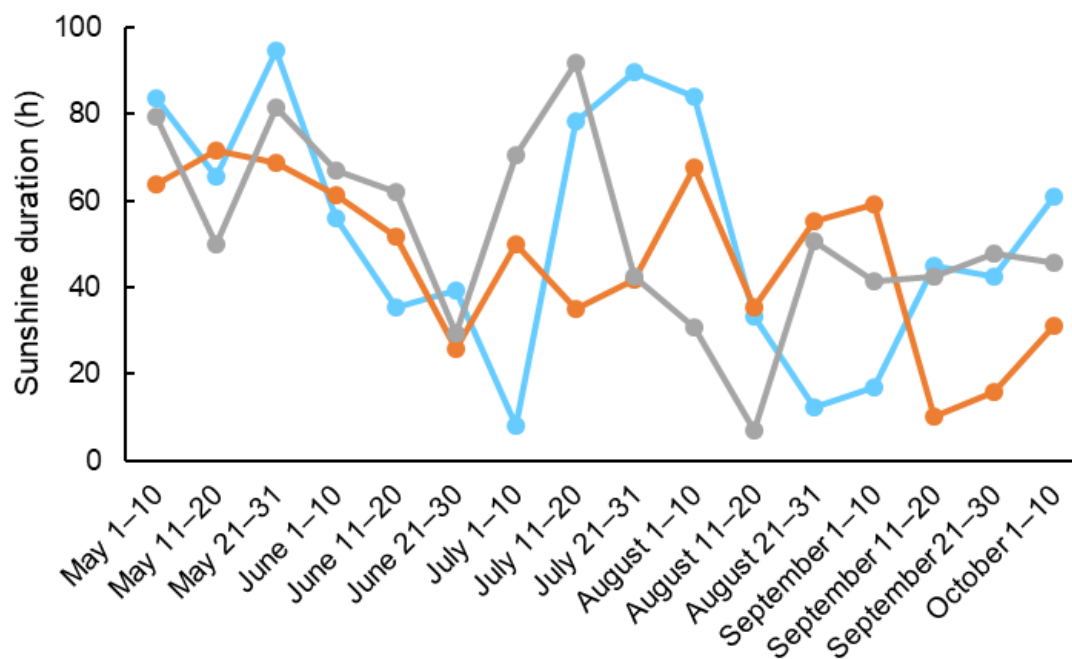

Supplement: S1 Fig — (A) Mean air temperature and precipitation. Light blue marker, mean air temperature in 2015; orange marker, mean air temperature in 2016; gray marker, mean air temperature in 2017. Yellow bar, precipitation in 2015; blue bar, precipitation in 2016; green bar, precipitation in 2017. (B) Sunlight duration. Light blue marker, sunlight duration in 2015; orange marker, sunlight duration in 2016; gray marker, sunlight duration in 2017. (PDF) [file pone.0221424.s001.pdf]

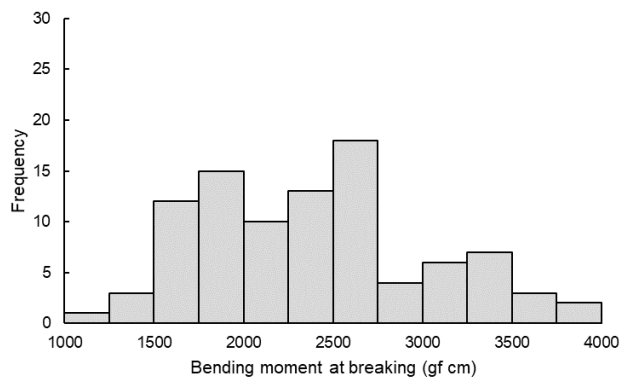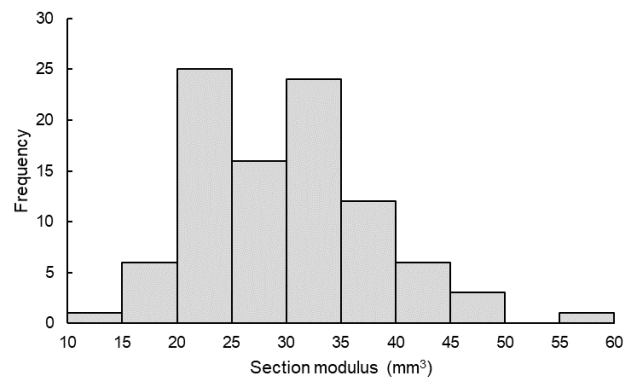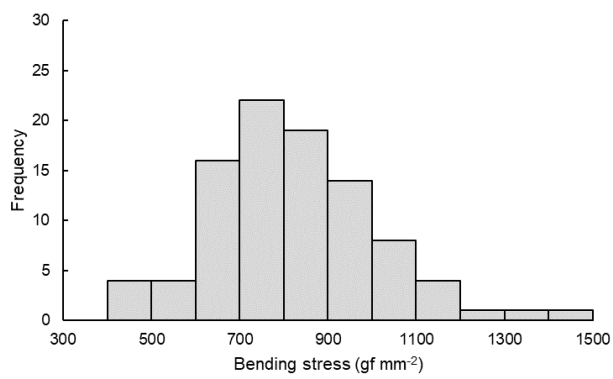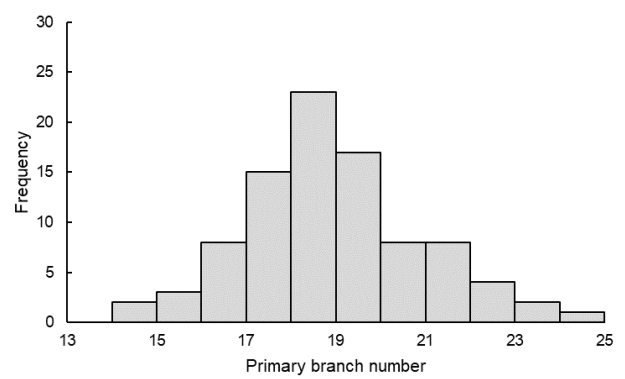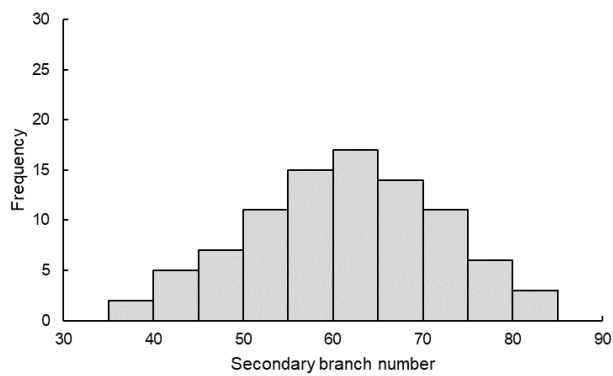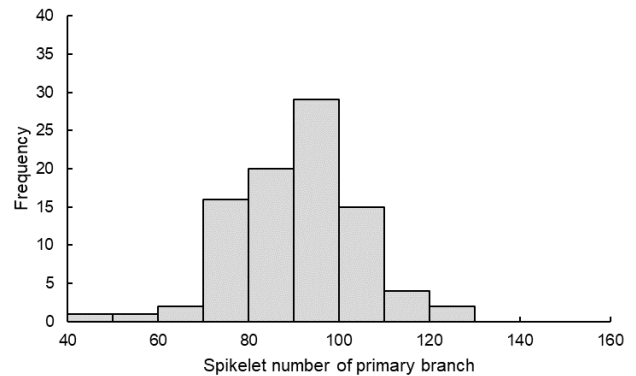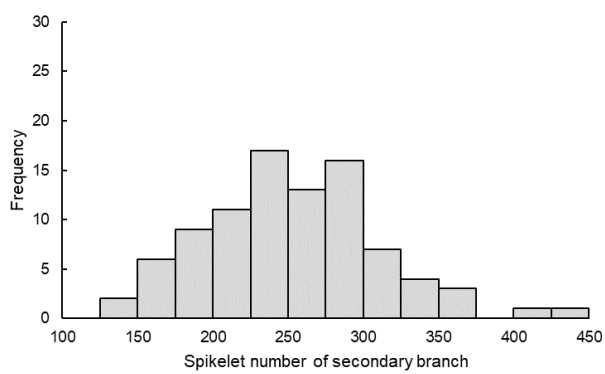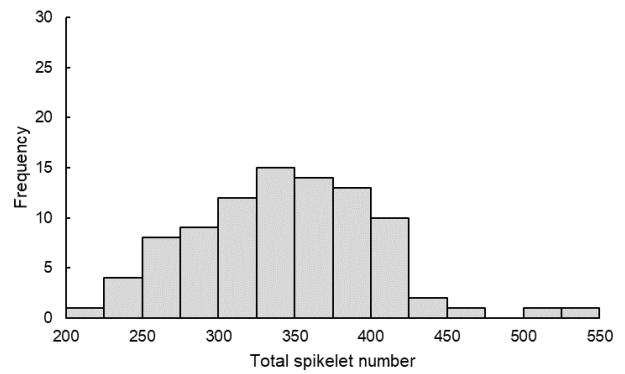

Supplement: S2 Fig — (PDF) [file pone.0221424.s002.pdf]

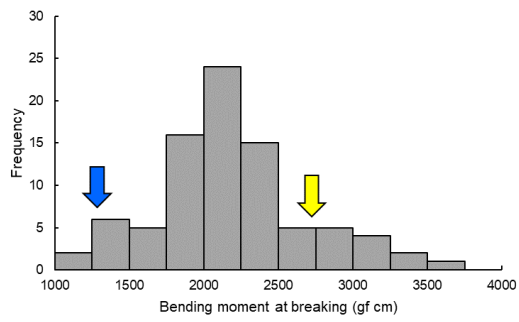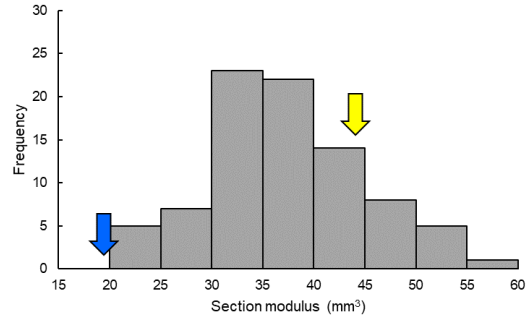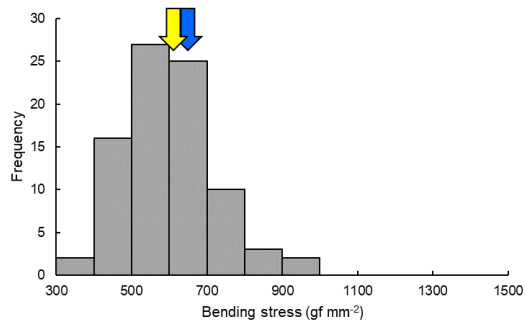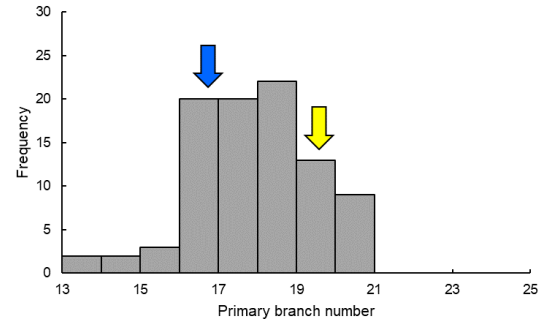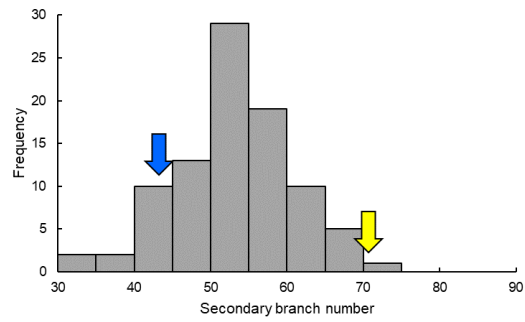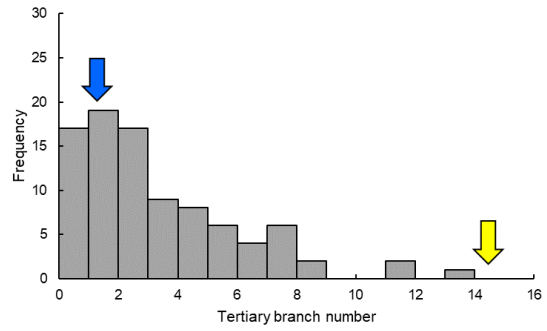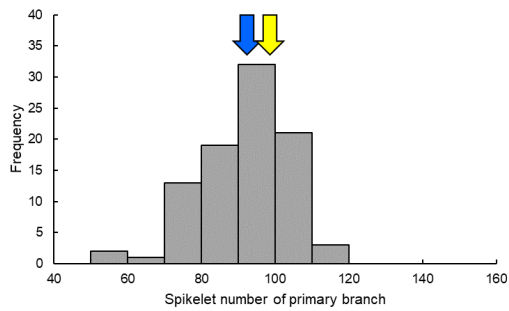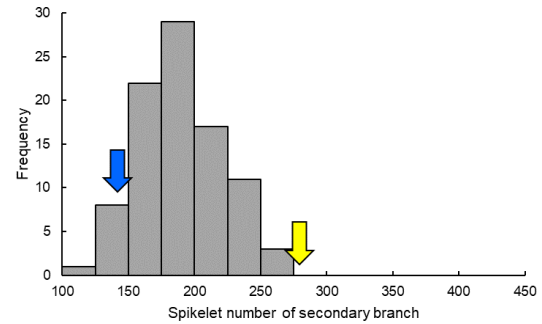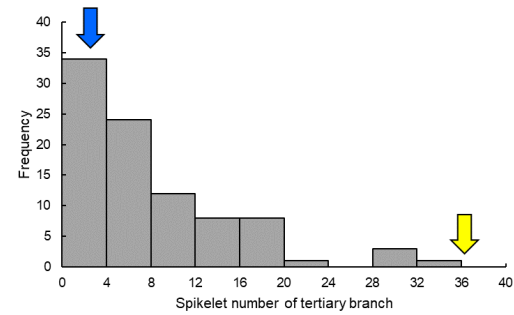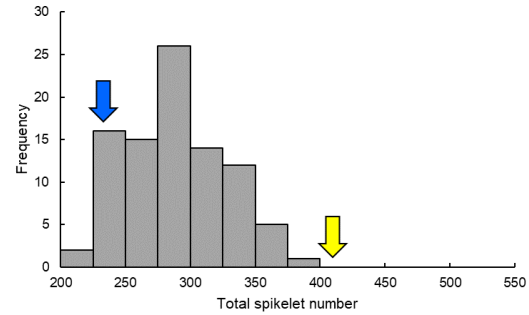

Supplement: S3 Fig — Blue and yellow arrows indicate mean of Takanari and Monster Rice 1, respectively. (PDF) [file pone.0221424.s003.pdf]

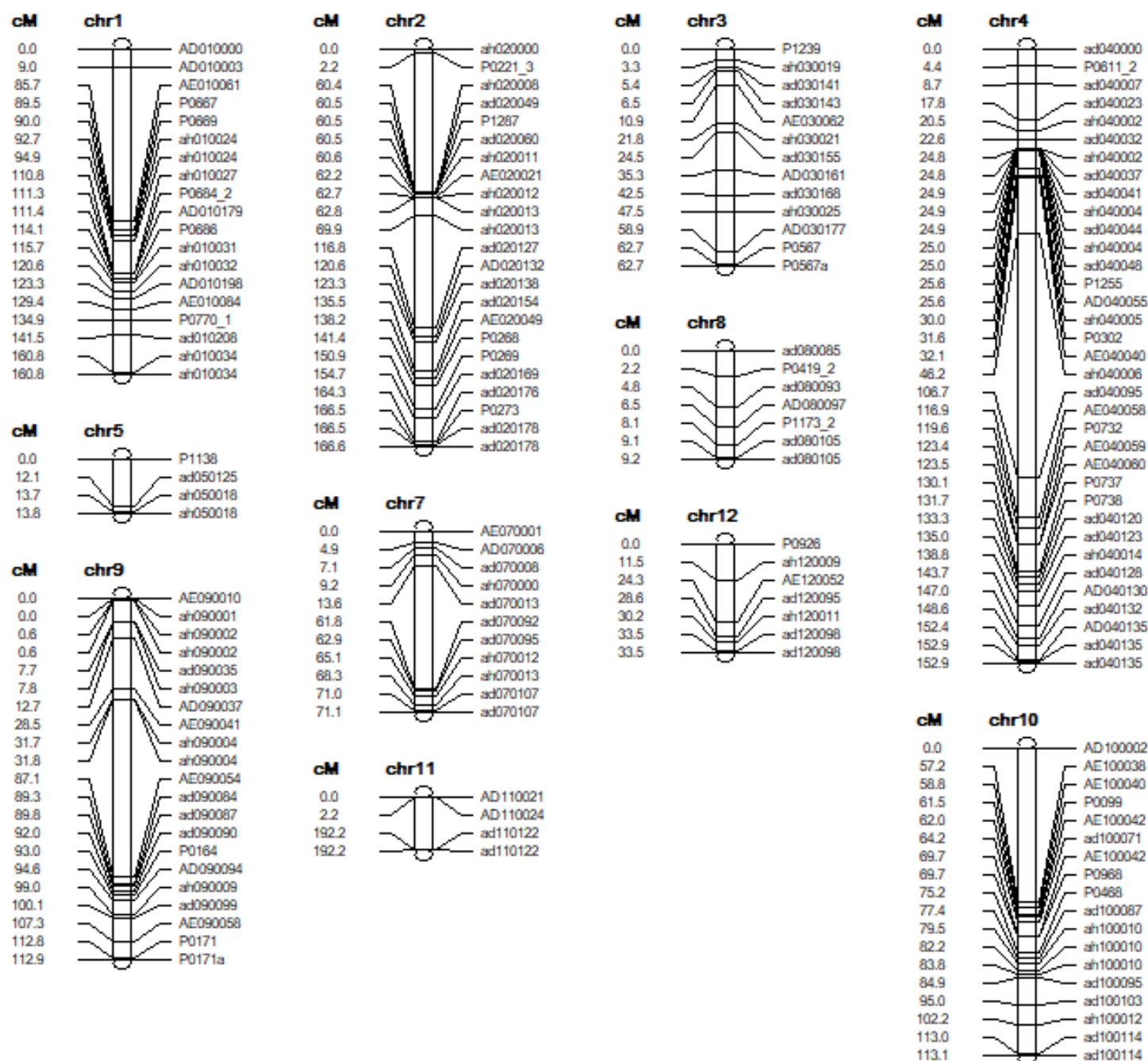

Supplement: S4 Fig — (PDF) [file pone.0221424.s004.pdf]

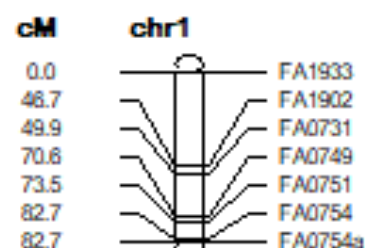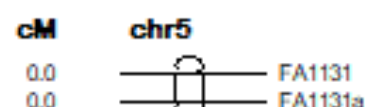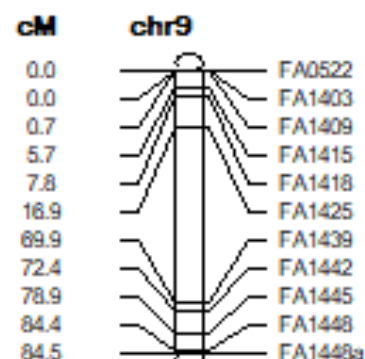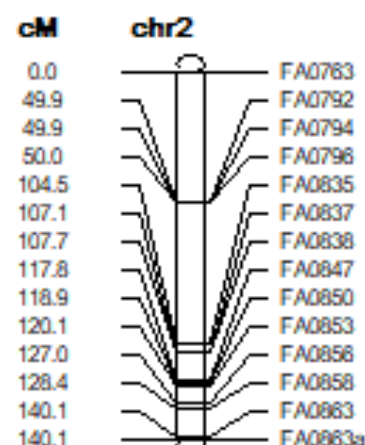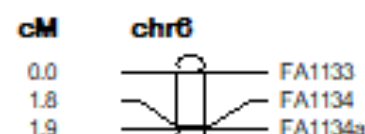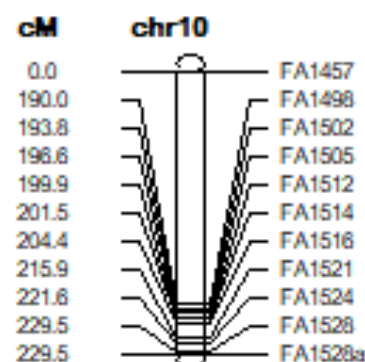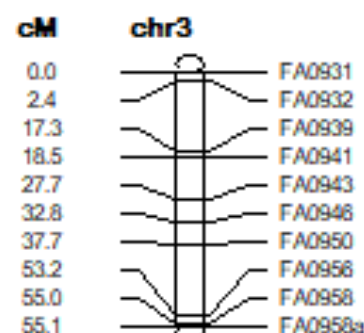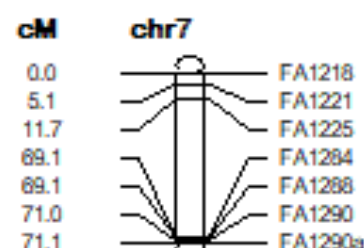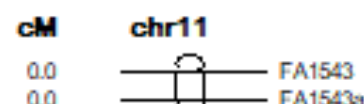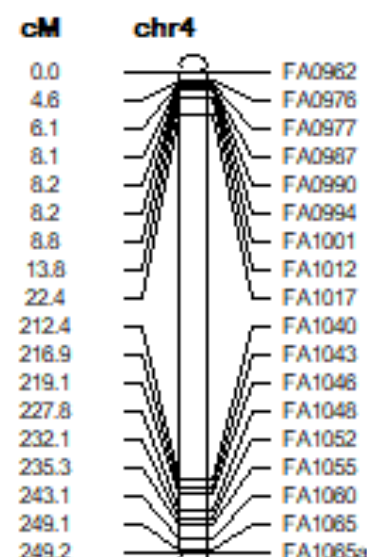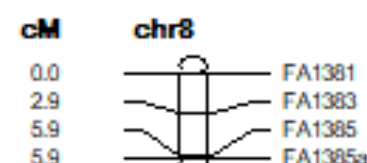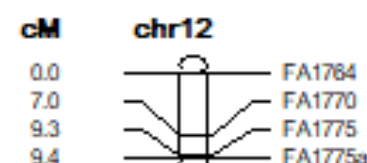

Supplement: S5 Fig — (PDF) [file pone.0221424.s005.pdf]

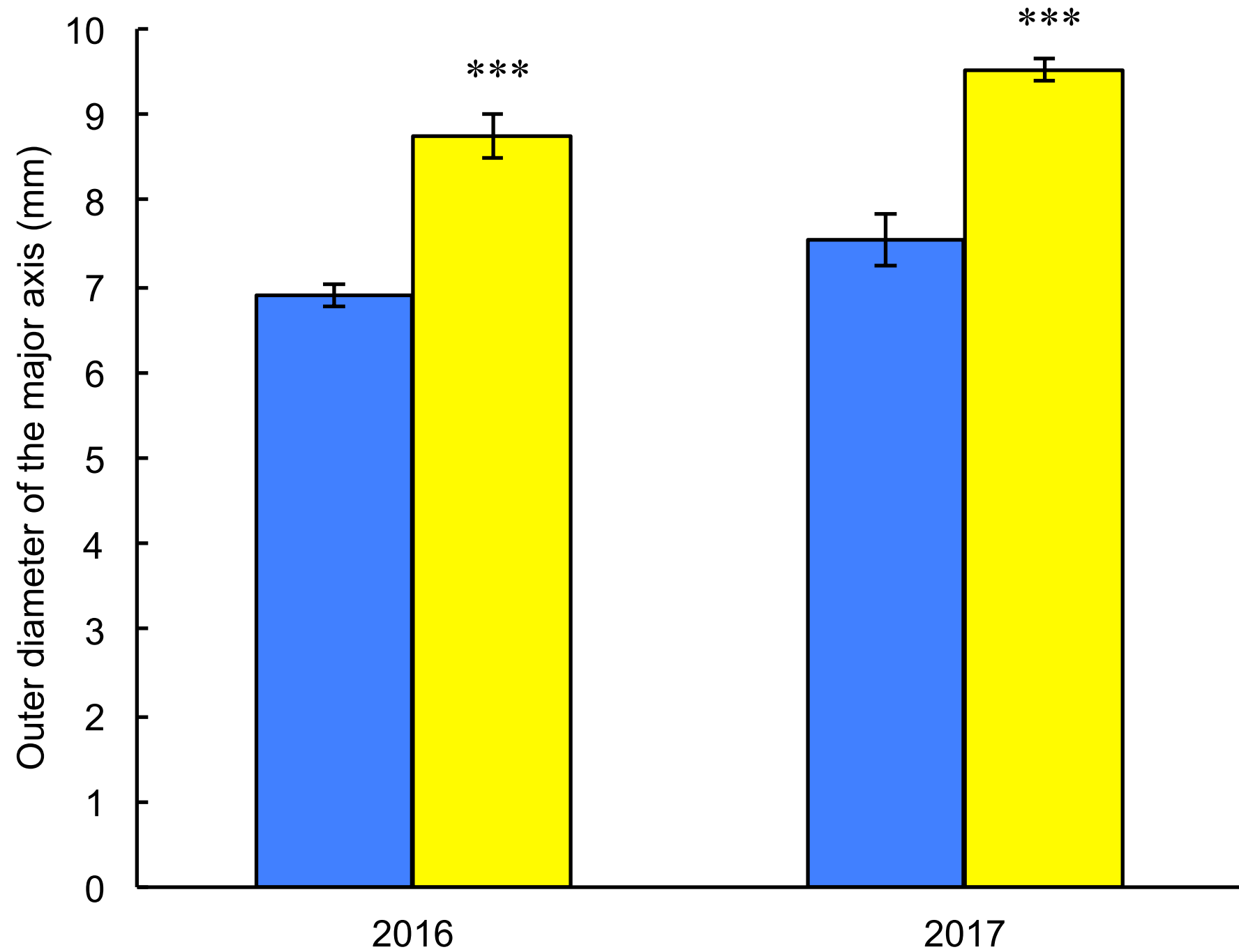

Supplement: S6 Fig — Blue and yellow bars indicate mean ± SD (n = 3) of Takanari and Monster Rice 1, respectively. Asterisks indicate significant difference between both cultivars: *** indicates P < 0.001 (t-test). (PDF) [file pone.0221424.s006.pdf]
